# B Lymphocytes as Targets of the Immunomodulatory Properties of Human Amniotic Mesenchymal Stromal Cells

**DOI:** 10.3389/fimmu.2020.01156

**Published:** 2020-06-09

**Authors:** Marta Magatti, Alice Masserdotti, Patrizia Bonassi Signoroni, Elsa Vertua, Francesca Romana Stefani, Antonietta Rosa Silini, Ornella Parolini

**Affiliations:** ^1^Centro di Ricerca E. Menni, Fondazione Poliambulanza Istituto Ospedaliero, Brescia, Italy; ^2^Department of Life Science and Public Health, Università Cattolica del Sacro Cuore, Rome, Italy

**Keywords:** mesenchymal stromal cells, amniotic membrane, placenta, B cells, plasma cells, plasmablast, conditioned medium, immunomodulation

## Abstract

Mesenchymal stromal cells (MSC) from the amniotic membrane of human term placenta (hAMSC), and the conditioned medium generated from their culture (CM-hAMSC) offer significant tools for their use in regenerative medicine mainly due to their immunomodulatory properties. Interestingly, hAMSC and their CM have been successfully exploited in preclinical disease models of inflammatory and autoimmune diseases where depletion or modulation of B cells have been indicated as an effective treatment, such as inflammatory bowel disease, lung fibrosis, would healing, collagen-induced arthritis, and multiple sclerosis. While the interactions between hAMSC or CM-hAMSC and T lymphocytes, monocytes, dendritic cells, and macrophages has been extensively explored, how they affect B lymphocytes remains unclear. Considering that B cells are key players in the adaptive immune response and are a central component of different diseases, in this study we investigated the *in vitro* properties of hAMSC and CM-hAMSC on B cells. We provide evidence that both hAMSC and CM-hAMSC strongly suppressed CpG-activated B-cell proliferation. Moreover, CM-hAMSC blocked B-cell differentiation, with an increase of the proportion of mature B cells, and a reduction of antibody secreting cell formation. We observed the strong inhibition of B cell terminal differentiation into CD138^+^ plasma cells, as further shown by a significant decrease of the expression of interferon regulatory factor 4 (*IRF-4*), PR/SET domain 1(*PRDM1*), and X-box binding protein 1 (*XBP-1*) genes. Our results point out that the mechanism by which CM-hAMSC impacts B cell proliferation and differentiation is mediated by secreted factors, and prostanoids are partially involved in these actions. Factors contained in the CM-hAMSC decreased the CpG-uptake sensors (CD205, CD14, and TLR9), suggesting that B cell stimulation was affected early on. CM-hAMSC also decreased the expression of interleukin-1 receptor-associated kinase (IRAK)-4, consequently inhibiting the entire CpG-induced downstream signaling pathway. Overall, these findings add insight into the mechanism of action of hAMSC and CM-hAMSC and are useful to better design their potential therapeutic application in B-cell mediated diseases.

## Introduction

Mesenchymal stromal cells (MSC) from the amniotic membrane of human term placenta (hAMSC), and the conditioned medium generated from their culture (CM-hAMSC) possess the ability to modulate inflammation (1). This intriguing property offers significant advantages for their use in the treatment of inflammatory and immunomediated diseases, and in the field of regenerative medicine (2). As a matter of fact, hAMSC and their CM have been successfully exploited in different preclinical disease models where inflammation occurs, including lung (3–6) and liver (7) fibrosis, would healing (8–10), collagen-induced arthritis (11, 12), multiple sclerosis (11), inflammatory bowel disease (11), colitis (11, 13), sepsis (11), traumatic brain injury (14), and Huntington's disease (15). We and others have demonstrated that hAMSC and CM-hAMSC suppress the *in vitro* proliferation, inflammatory cytokine production, and functions of T lymphocytes (16, 17), monocytes (10), dendritic cells (18), macrophages (10), and natural killer cells (19), and are able to induce a phenotype and functional switch of monocytes toward macrophages with anti-inflammatory pro-regenerative M2-like features (10, 17), and also support the expansion of regulatory T cells (16, 17). These *in vitro* immunomodulatory actions have been confirmed in preclinical studies (4, 11, 13). However, studies which address how hAMSC or their CM affect B-cell functions are lacking.

Together with T cells, B cells are key players in the adaptive immune response, they are potent antigen presenting cells that can produce both pro- and anti-inflammatory cytokines, and have the capacity to generate terminally differentiated antibody-secreting plasma cells (20–22). Thus, B cells represent important targets for the treatment of multiple autoimmune disorders (23), for the induction of graft survival (24), or for the treatment of skin and lung fibrosis (25, 26), and can act as powerful modulators of tissue regeneration (27, 28). There is evidence of the ability of MSC to interact with B cells, however controversial effects have been described (29, 30). Indeed, several authors have demonstrated that MSC from bone marrow (BM-MSC) and adipose tissue (AT-MSC) (31, 32) strongly inhibit B-cell proliferation (31, 33–36), but this antiproliferative capacity has not been confirmed by others (37–39). In addition, although a significant inhibition of plasma cell formation and decrease of immunoglobulin production has been shown in some studies (31–36), an increased differentiation into plasma cells with increased Ig production has been observed in others (38, 39). Currently, there are only a few studies with placenta-derived MSC, which are referred to MSC isolated from umbilical cord (38, 40) or Wharton's jelly (41). Moreover, these studies were limited to the investigation of only mouse B cells (40), or cell lines (Burkitt's lymphoma cell lines) (41), or only analyzed the effect of placenta MSC on the proliferation of B lymphocytes (42).

Therefore, in this study we investigated the *in vitro* properties of hAMSC and CM-hAMSC on B-cell proliferation and differentiation. We analyzed the possible mechanism of action by which CM-hAMSC acts on B cells by examining the signaling pathways involved in B-cell activation and the genes responsible for plasma cell generation. Finally, since we have previously shown that prostanoids are partially responsible for the hAMSC-induced inhibition of T-cell proliferation (43), we investigated whether they could be involved in the effects observed on B cells.

## Materials and Methods

### Ethics Statement

The collection of human peripheral blood mononuclear cells (PBMC) for research purposes was approved by the Regional Departments of Transfusion Medicine (Rif. 523, July 7, 2016). PBMC were obtained from healthy adult donors (*n* = 10) and provided by Center of Immune Transfusion of Spedali Civili of Brescia, Italy. Human term placentas (*n* = 15), recovered from healthy women after vaginal delivery or cesarean section at term, were provided by the Department of Obstetrics and Gynecology of Fondazione Poliambulanza-Istituto Ospedaliero of Brescia, Italy. Samples were collected after obtaining informed written consent according to the guidelines set by the *Comitato Etico Provinciale* of Brescia, Italy number NP 2243 (19/01/2016).

### Isolation of Human Amniotic Mesenchymal Stromal Cells and Preparation of Conditioned Medium

Placentas were processed immediately after collection and cells were isolated and directly used. Specifically, human amniotic mesenchymal stromal cells (hAMSC) were obtained from the mesenchymal region of the amniotic membrane as previously described (44). Conditioned medium was generated by culturing hAMSC (CM-hAMSC) for 5 days in 24-well plates (Corning, NY, USA) at a density of 5 × 10^5^ cells/well in 0.5 ml of Ultraculture complete medium, composed of Ultraculture medium (Sigma-Aldrich, St Louis, MO, USA), supplemented with 2 mM L-glutamine (Sigma-Aldrich), and 100 U/ml penicillin and 100 mg/ml streptomycin (both from Sigma-Aldrich) as described (43). To obtain CM devoid of prostanoids (CM-hAMSC/PG), hAMSC were cultured in Ultraculture complete medium supplemented with 10 μM indomethacin (Sigma-Aldrich), a cyclooxygenase inhibitor (10). At the end of culture, CM-hAMSC and CM-hAMSC/PG were collected, centrifuged at 300 x g, filtered through a 0.8 μm sterile filter (Sartorius Stedim, Florence, Italy), and kept frozen at −80°C until use. Each experiment was performed by pooling together conditioned medium obtained from 3 to 4 different hAMSC donors.

### Isolation of PBMC and B Cells

PBMC were separated from sodium citrate whole blood through density gradient centrifugation (Histopaque, Sigma-Aldrich), were frozen in fetal bovine serum (FBS, Sigma-Aldrich) with 10% DMSO (Sigma-Aldrich) and stored in liquid nitrogen. B cells were isolated from PBMC (*n* = 5) by negative selection using Pan B cell isolation kit and MACS® separation columns (Miltenyi Biotec, Bergisch Gladbach, Germany), according to the manufacturer's instructions. The purity of the B cell population was confirmed with flow cytometry by CD19 expression which resulted >85–90% of the total cells recovered.

### Activation of B Cells and Co-culture With hAMSC or CM-hAMSC

PBMC or enriched B cells were labeled with the intracellular fluorescent dye, carboxyfluorescein diacetate succinimidyl ester (CFSE, ThermoFisher) to monitor cell division. Briefly, 1 × 10^6^ cells/mL were resuspended in PBS-0.5% FBS and incubated with 1 μM CFSE for 4 min at 37°C. The cells were then washed three times in cold RPMI complete medium, composed of RPMI 1,640 medium supplemented with 10% heat-inactivated fetal bovine serum (FBS), 1% penicillin and streptomycin, and 1% L-glutamine (all from Sigma Aldrich, St. Louis, Missouri, USA), and 2 × 10^5^ PBMC or 1 × 10^5^ B cells were subsequently plated in 96 well-tissue culture plate in Ultraculture complete medium (Sigma-Aldrich). B cell proliferation was induced by stimulating cells for 6 days with 2.5 μg/ml CpG-ODN 2006 (Aurogene Srl, Rome, Italy). Activated PBMC or B cells were cultured in the presence of 100 μl of CM-hAMSC or in contact with 1 × 10^5^ hAMSC, that were plated the day before in RPMI complete medium, left to adhere overnight, and γ-irradiated at 30Gy to block their proliferation. The amount of CM-hAMSC and hAMSC used in the experiments was previously determined by culturing PBMC in the presence of different amounts of CM-hAMSC (100, 50, 10 μl/well) or different concentrations of hAMSC (1 × 10^5^, 0.5 × 10^5^, 0.1 × 10^5^). The final volume of each well was 200 μl. In all experiments activated PBMC or B cells cultured alone were used as control.

### Flow Cytometry Analysis of B Cell Proliferation and Subsets

Flow cytometry analysis of B cells was performed after 6 days of co-culture with hAMSC or CM-hAMSC, as described above. Dead cells were excluded by eBioscience™ Fixable Viability Dye eFluor™ 780 (Thermo Fisher Scientific, Waltham, MA USA) staining, according to the manufacturer's instructions. B cell subsets were identified by staining for 20 min at 4°C with the appropriate combination of fluorochrome-conjugated anti-human antibodies: CD19 BV421 (clone HIB19, used at final concentration of 1:200) or CD19 BB700 (SJ25C1, 1:200), CD3 BV510 (UCHT1, 1:100), CD14 BV510 (MΦP9, 1:200), CD38 PE (HB7, 1:150), CD27 PE-Cy7 (M-T271, 1:100), CD138 APC (44F9,1:67), all from BD Biosciences except for CD138 that was from Miltenyi. After washing in stain buffer, consisting of 0.02% sodium azide and 0.1% bovine serum albumin in PBS (Sigma-Aldrich), the cells were acquired on FACSAria III (BD Biosciences). B cell proliferation was inferred by analysis of CFSE dilution, and calculated as percentage of CD19^+^ proliferating cells. Raw data were analyzed using FCS express v5.0 (*DeNovo* Software, Los Angeles, CA, USA).

### Flow Cytometry Analysis of CpG Sensors and CpG-Induced B-Cell Signaling Transduction

2 × 10^5^ PBMC were plated in 96 well-tissue culture plate in Ultraculture complete medium (Sigma-Aldrich) and stimulated with 2.5 μg/ml CpG-ODN 2006 (Aurogene Srl). Activated PBMC were cultured alone or in the presence of 100 μl of CM-hAMSC in a final volume of 200 μl. After 2 days of culture, the effect of CM-hAMSC on CpG sensors (CD205, CD14, TLR9) expressed on B cells was assessed by staining the cells for 20 min at 4°C with CD19 BV421 (HIB19, 1:200), CD3 BV510 (UCHT1, 1:100), CD14 A647 (MΦP9, 1:200), CD205 (DEC-205) BB700 (MMRI-7, 1:100), all from BD Biosciences. After a washing step, the cells were fixed and permeabilized with Cytofix/cytoperm solution (BD Biosciences) for 20 min at 4°C, washed with 1 × BD perm/wash buffer solution (BD Biosciences), and stained for 20 min at 4°C with specific antibody against CD289 (TLR9) PE (eB72-1665, 1:20) and CD14 PE (MΦP9, 1:50). Finally, the cells were washed and acquired. To analyze CpG-induced signaling transduction, cells were collected and 2 × 10^6^ cells/ml stimulated with 2.5 μg/ml CpG-ODN 2006 (Aurogene Srl) for 1 h. In order to preserve phosphorylation state, the cells were fixed immediately after the stimulation period by adding 1.5% methanol-free formaldehyde (ThermoFisher) for 10 min at room temperature (RT). Then, the cells were washed and stained for 25 min at 4°C with CD19 BV421 (HIB19, 1:200), CD14 BV510 (MΦP9, 1:200), CD3 FITC (UCHT1, 1:100), all from BD Biosciences. After a permeabilization step in cold 90% methanol for 15 min at 4°C in the dark, the cells were stained, protected from light, for 1h at RT with BD Phosflow™ (BD Biosciences) fluorescently conjugated antibodies against: IRAK-4 PE (clone L29–525, 1:20), p38 MAPK pT180/pY182 Alexa Fluor® 647 (36/p38 pT180/pY182, 1:20), NF-κB p65 (pS529) Alexa Fluor® 647 (K10-895.12.50, 1:40), JNK (pT183/pY185) Alexa Fluor® 647 (N9-66, 1:40), ERK1/2 (pT202/pY204) PerCP-Cy™5.5 (20A, 1:20), PDPK1 (pS241) PE (J66-653.44.17, 1:20), Akt (pS473) PE-CF594 (M89-61, 1:40), Stat1 (pY701) PE (4a, 1:33), Stat5 (pY694) PE-Cy7 [47/Stat5(pY694), 1:20]. Finally, the cells were washed and acquired on FACSAria III (BD Biosciences) and FCS express v. 5.0 (*DeNovo* Software, Los Angeles, CA, USA) was used for data analysis. All the washing steps were performed with stain buffer unless stated otherwise.

### Quantitative Real-Time PCR

2 × 10^5^ PBMC were plated in 96 well-tissue culture plates in Ultraculture (Lonza) complete medium and stimulated with 2.5 μg/ml CpG-ODN 2006 (Aurogene Srl). Activated PBMC were cultured alone or in the presence of 100 μl of CM-hAMSC in a final volume of 200 μl. PBMC were collected at days 3, 4, and 5 after culture, and washed with PBS (Sigma-Aldrich). Total RNA was extracted using EZ1 RNA cell Mini Kit protocol (Qiagen, Frederick, MD, USA), in a BioRobot EZ1 Advanced XL Workstation. The iScript Advanced cDNA Synthesis Kit for RT-qPCR (Biorad) was used for cDNA synthesis, and quantitative real-time PCR was performed using a Biorad instrument CFX96 (Biorad) with technical triplicates in 96-well plates. SsoAdvanced Universal SYBR Green Supermix (Biorad) was used following manufacturer's instructions in a total volume of 20 μl. The real-time PCR cycling program was as follows: 30 s at 95°C, 40 cycles of 10 s at 95°C, 20 s at 58°C. Data were analyzed with the GeneGlobe Data Analysis Center (Qiagen). Primer sequences, listed in Table 1, were purchased from Sigma-Aldrich. The expression level of each mRNA was normalized to the level of the endogenous controls β*-ACTIN*.

**Table 1:** Primer sequences of genes analyzed by quantitative real-time PCR.

	**Primer sequence (Forward)**	**Primer sequence (Reverse)**
*SDC1*	5′-GCCGCAAATTGTGGCTACT-3′	5′-GCTGCGTGTCCTTCCAAGT-3′
*PAX5*	5′-GGAGGAGTGAATCAGCTTGG-3′	5′-GGCTTGATGCTTCCTGTCTC-3′
*BCL6*	5′-GAGAAGCCCTATCCCTGTGA-3′	5′-TGCACCTTGGTG TTGGTGAT-3′
*XBP1*	5′-CCTGGTTGCTGAAGAGGAGG-3′	5′-CCATGGGGAGATGTTCTGGAG-3′
*IRF4*	5′-CTACACCATGACAACGCCTTACC-3′	5′-GGCTGATCCGGGACGTAGT-3′
*PRDM1*	5′-TCAAACTCAGCCTCTGTCCA-3′	5′-TCCAGCACTGTGAGGTTTCA-3′
*β-ACTIN*	5′-GGATGCAGAAGGAGATCACTG-3′	5′-CGATCCACACGGAGTACTTG-3′

### Flow Cytometry Analysis of Transcription Factors

PBMC were plated and stimulated with CpG-ODN 2006 (Aurogene Srl) alone or in the presence of 100 μl of CM-hAMSC as described above. After 5 days of culture, cells were collected and protein expression of the transcription factors BCL6, PAX-5, IRF-4, and BLIMP-1 was analyzed by flow cytometry. After exclusion of dead cells by eBioscience™ Fixable Viability Dye eFluor™ 780 (Thermo Fisher Scientific) staining, cells were stained for 20 min at 4°C with CD19 BB700 (SJ25C1, 1:200), CD3 BV510 (UCHT1, 1:100), CD14 BV510 (MΦP9, 1:200), CD27 PE-Cy7 (M-T271, 1:100), all from BD Biosciences. After washing in stain buffer, the cells were fixed and permeabilized with Transcription Factor Buffer Set (BD Biosciences) for 40 min at 4°C, according to the manufacturer's instructions. Then, cells were stained for 40 min at 4°C with specific antibody against BCL6 BV421 (K112-91, 1:30), PAX-5 PE (1H9, 1:100), IRF-4 PE (Q9–343, 1:100), BLIMP-1 Alexa Fluor® 647 (6D3, 1:100). Finally, the cells were washed with Transcription Factor Buffer Set (BD Biosciences), acquired on a FACSAria III (BD Biosciences), and analyzed using FCS express v5.0 (*DeNovo* Software, Los Angeles, CA, USA).

### Analysis of Cytokine and Chemokines

PBMC were plated and stimulated with CpG-ODN 2006 (Aurogene Srl) alone or in the presence of 100 μl of CM-hAMSC as described above. After 6 days of culture, the supernatant was collected and stored at −80 °C. The amount of human interleukin (IL)-2, IL-5, IL-8, IL-10, IL-21, interferon γ-induced protein (IP-10/CXCL10), monocyte chemoattractant protein-1 (MCP-1/CCL2), granulocyte-colony stimulating factor (G-CSF), granulocyte-macrophage colony-stimulating factor (GM-CSF), and macrophage inflammatory proteins (MIP)-1α was determined by a multiplex bead-based immunoassay (BD CBA Flex Set system from BD Biosciences). Samples were processed according to the manufacturer's instructions, acquired on a FACSAria III (BD Biosciences), and analyzed with the FCAP Array software (BD Biosciences).

### Statistical Analysis

**Figure 1:**
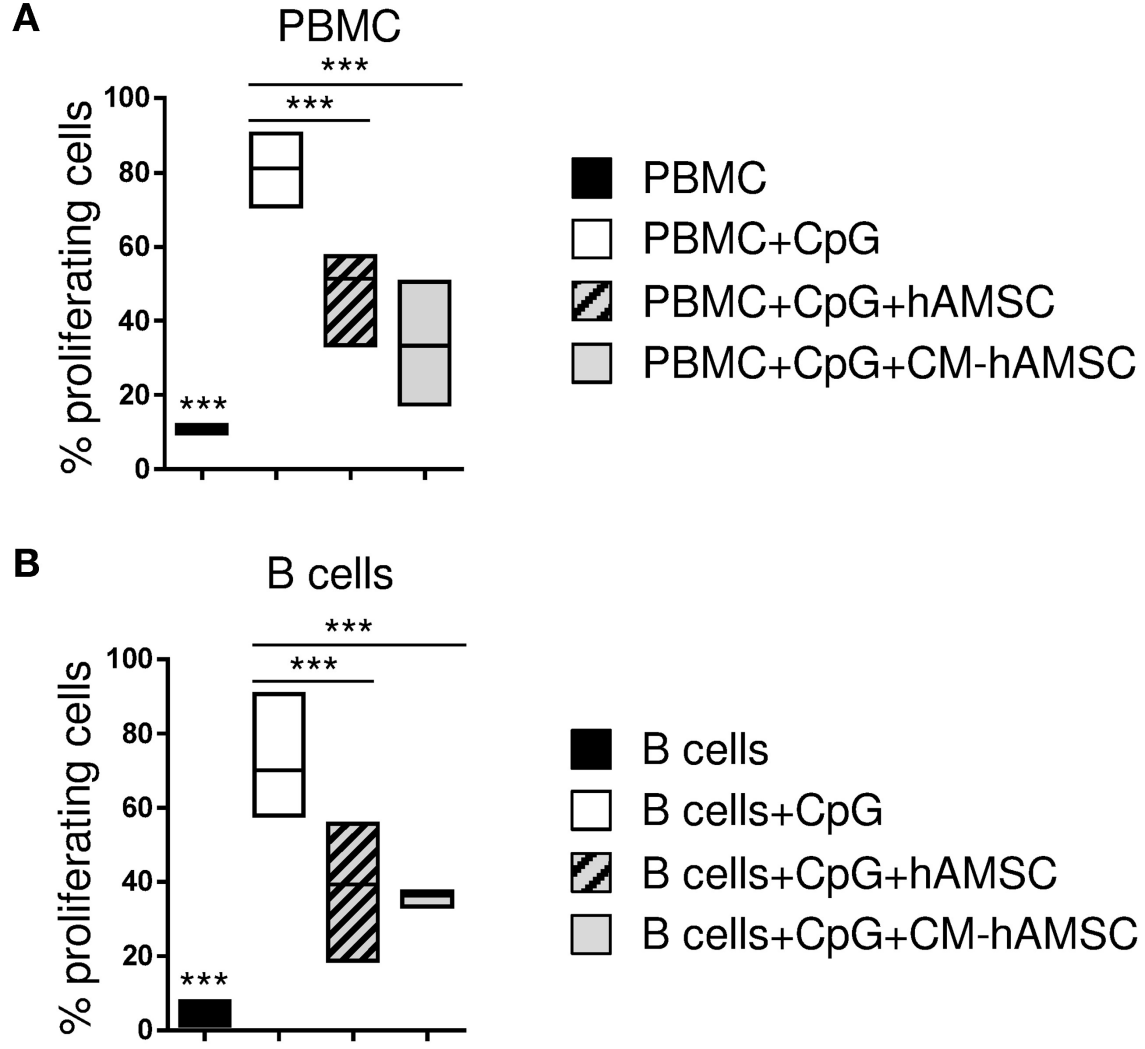
Peripheral blood mononuclear cells (PBMC) **(A)** or enriched B cells **(B)** were labeled with carboxy-fluorescein diacetate, succinimidyl ester (CFSE) fluorescence. PBMC or B cells were stimulated *in vitro* with CpG to induce B-cell proliferation (white bar, control) and cultured in contact with 1 × 10^5^ hAMSC (dashed gray bar) or in presence of 100 μl of CM-hAMSC (gray bar). Unstimulated PBMC or B cells (black bar) were used as controls. After 6 days of culture, B-cell proliferation was measured with flow cytometry by the analysis of CFSE dilution, and calculated as percentage of CD19^+^ proliferating cells. Floating bars (min to max) with line at median of proliferation from 7 **(A)** and 5 **(B)** individual experiments is reported. ***p < 0.001 *vs*. PBMC+CpG.

**Figure 2:**
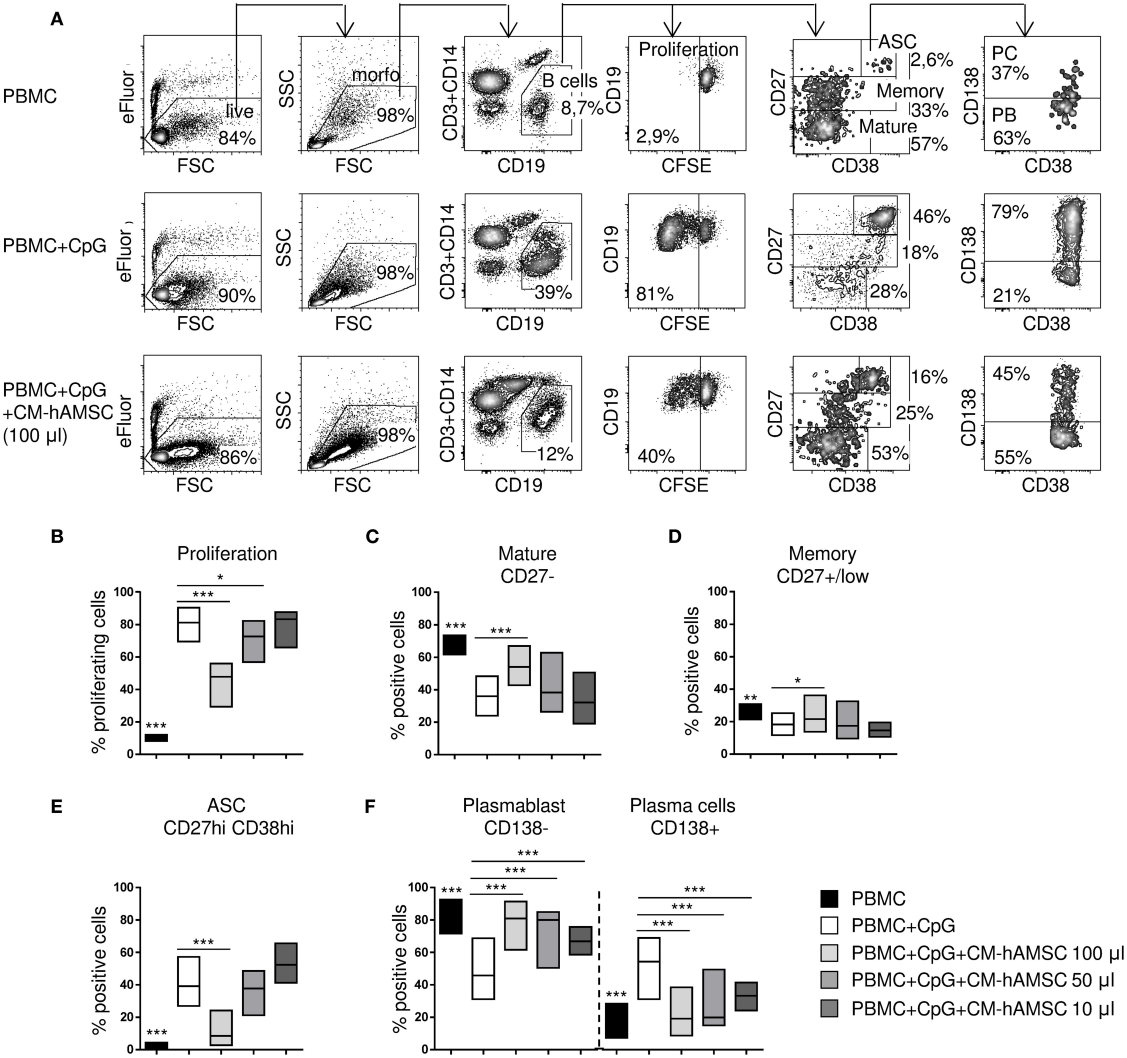
PBMC were labeled with carboxy-fluorescein diacetate, succinimidyl ester (CFSE) fluorescence, stimulated *in vitro* with CpG and cultured in presence of different concentrations of CM-hAMSC. Unstimulated PBMC were used as controls. After 6 days of culture, B-cell proliferation and B-cell subsets were measured with flow cytometry. Representative results and gating strategy applied are shown **(A)**. After removal of dead cells by eBioscience™ Fixable Viability Dye eFluor™ 780 exclusion and morphological (FSC, forward scatter and SSC, side scatter) gates, B cells were identified as CD19^+^ (and CD3^−^CD14^−^) cells. Then, B-cell proliferation was measured by the analysis of CFSE dilution, and calculated as percentage of CD19^+^ proliferating cells. Mature B cells, memory B cells, and antibody secreting cells (ASC), were herein identified in the gate of CD19^+^ cells as CD27^−^, CD27^+^/low, and CD27^hi^CD38^hi^, respectively. Finally, in the gate of ASC (CD19^+^CD27^hi^CD38^hi^), plasma cells (PC) were identified as CD138^+^ and plasmablast (PB) as CD138^−^. The percentage of positive cells in each gate is reported **(A)**. Floating bars (min to max) with line at median from at least 9 individual experiments is reported for the percentage of proliferating B cells **(B)**, mature B cells (CD19^+^CD27^−^) **(C)**, memory B cells (CD19^+^CD27^+^/low) **(D)**, ASC (CD19^+^CD27^hi^CD38^hi^) **(E)**, and the proportion of plasmablasts (CD138^−^) and plasma cells (CD138^+^) **(F)**. *p < 0.05, ***p < 0.001 *vs*. PBMC+CpG.

**Figure 3:**
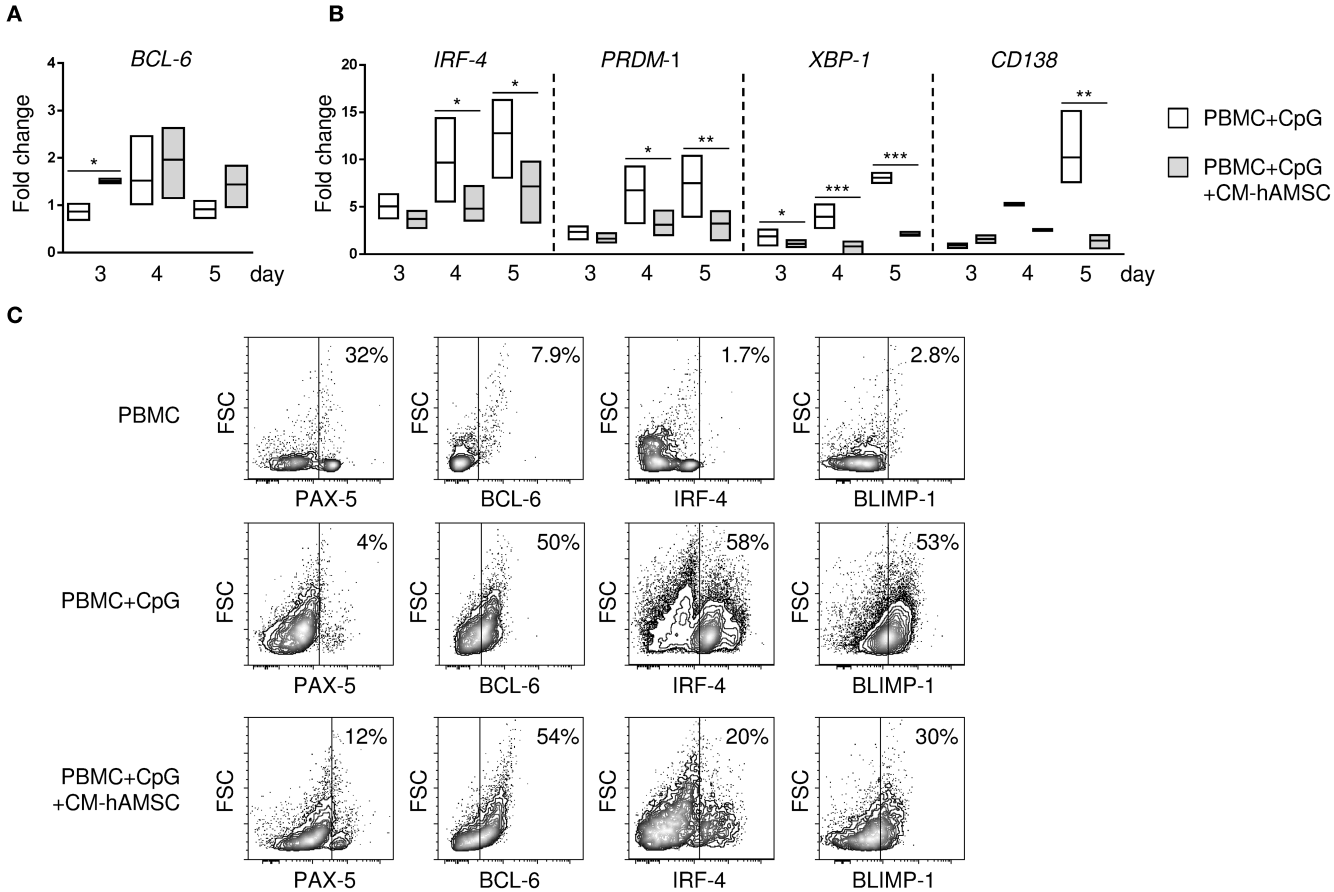
PBMC were stimulated *in vitro* with CpG in absence or presence of 100 μl CM-hAMSC. After 3, 4, and 5 days of culture, PBMC were collected, and quantitative real-time PCR was performed for the analysis of B-cell CLL/lymphoma 6 (*BCL6*) **(A)**, and interferon regulatory factor 4 (*IRF-4*), PR/SET domain 1 (*PRDM1*), X-box binding protein 1 (*XBP-1*), and syndecan 1 (*SDC1*/*CD138*) **(B)**. The fold change in mRNA expression in PBMC+CpG and PBMC+CpG+CM-hAMSC vs. unstimulated PBMC collected at day 3 is reported. Data are expressed as floating bars with line at median from at least 3 independent experiments, *p < 0.05, **p < 0.01, ***p < 0.001 *vs*. PBMC+CpG. The protein expression of the transcription factors BCL6, paired box 5 (PAX-5), B lymphocyte induced maturation protein-1 (BLIMP-1), and IRF-4 was evaluated by flow cytometry. Representative results of 4 independent experiments are shown **(C)**.

One-way analysis of variance (ANOVA) was used to evaluate significance, and the Holm-Sidak correction for multiple comparison was applied for data presented in Figures 1, 2, **6**, **7**. Two-way ANOVA with Sidak correction was applied for data presented in Figure 3. A paired *t-*test was used for data presented in **Figure 5**. P < 0.05 was considered to be statistically significant. Statistical analyses were performed using GraphPad Prism software 6 (GraphPad Software, La Jolla, CA, USA).

## Results

### Impact of hAMSC and CM-hAMSC on B Cell Proliferation

CpG ODN-2006 (from now on referred to as CpG) is a synthetic oligonucleotide that induces B cell proliferation and differentiation (45, 46). As expected, we observed that PBMC stimulated with CpG showed a high proliferation of CD19^+^ B-cells while those cultured in the absence of CpG did not proliferate (Figure 1A). CpG-activated PBMC co-cultured with hAMSC showed a significant reduction of CD19^+^ B-cell proliferation (Figure 1A). To determine whether the inhibitory effect of hAMSC on B cell proliferation was contact-dependent or mediated by secreted factors, we used the conditioned medium obtained from hAMSC culture (CM-hAMSC). As shown in Figure 1A, CM-hAMSC alone was sufficient to suppress CD19^+^ B cell proliferation, implying that hAMSC do not require cell-to-cell contact to carry out this particular function. Interestingly, similar results were obtained with purified B-cells (Figure 1B), indicating that inhibition of proliferation is exerted directly by hAMSC and CM-hAMSC on B cells and is likely not mediated by a third-party subset.

### Impact of CM-hAMSC on B Cell Sub-Populations

Having demonstrated that cell-to-cell contact is not necessary for B cell inhibition, we performed subsequent experiments with CM-hAMSC. First, different doses of CM-hAMSC corresponding to 1/2 (100 μl), 1/4 (50 μl), and 1/20 (10 μl) of the total volume of culture medium were evaluated. Inhibition of proliferation by CM-hAMSC was dose-dependent, indeed the capacity of CM-hAMSC to suppress B-cell proliferation increased with the dose (Figure 2B). We next sought to investigate the effect of CM-hAMSC on the principal B-cell subsets: mature B cells, memory B cells, and antibody-secreting cells (ASC), based on the different expression of CD27 and CD38 on CD19^+^ B cells (47, 48) (Figure 2A). Unactivated peripheral blood B cells mostly consisted of CD27^−^ mature B cells and CD27^+^/low memory B cells. *In vitro* CpG stimulation induces both mature and memory B cells to proliferate and to differentiate into ASC (45, 49). During proliferation and differentiation, CD27 (as well as CD38) is highly up-regulated, and increased expression of CD27 and CD38 were shown to correlate with antibody secretion (45, 49). Therefore, after CpG activation, CD27-positive cells represent heterogeneous population of B cells, in which CD27^hi^CD38^hi^ cells are those with the higher frequency of ASC (50, 51). In contrast, non-differentiated CD27^+^/low B cells were shown to preserve features of non-secreting memory B cells (50, 51). Thus, mature B cells, memory B cells, and ASC, were herein phenotypically identified in the CD19^+^ gate as CD27^−^, CD27^+^/low, and CD27^hi^CD38^hi^, respectively (Figure 2A).

Compared to unactivated PBMC, CpG-stimulated PBMC showed a strongly percentage of CD27^hi^CD38^hi^ ASC (Figure 2E) and a lower proportion of CD27^−^ mature (Figure 2C) and CD27^+^/low memory (Figure 2D) B cells. When CpG-stimulation was performed in the presence of the highest concentration of CM-hAMSC (100 μl), we observed an increase of mature B cells (Figure 2C), and memory B cells (Figure 2D), indicating the inhibition of B cell differentiation. In fact, in the presence of the highest concentration of CM-hAMSC, the differentiation of ASC was strongly and significantly suppressed, while decreasing concentrations did not display any effect on ASC formation (Figure 2E). There are currently two recognized subsets of ASC: plasmablasts and plasma cells, which can be discriminated based on their expression of the proteoglycan CD138, a hallmark of terminally differentiated plasma cells (48, 52). The few ASC found in the unactivated PBMC are mainly composed of CD138^−^ plasmablasts. On the other hand, CD138^+^ plasma cells represent the majority of the CpG-activated PBMC (Figure 2F). When activation of PBMC was performed in the presence of CM-hAMSC, we observed a higher presence of plasmablasts (and consequently a lower percentage of plasma cells) compared to the control PBMC stimulated in absence of CM-hAMSC. It is worth noting that, conversely to what we observed with CD19^+^ cell proliferation and ASC formation, a significant inhibition of plasma cells was also obtained at low concentrations of CM-hAMSC (50 and 10 μl) (Figure 2F). These results indicate that CM-hAMSC contains different factors able to strongly interfere not only with B-cell proliferation but also with their differentiation.

### Effects of CM-hAMSC on B-Cell Gene Expression

The differentiation of B cells into terminally differentiated plasma cells is under the control of several transcription factors. To better understand the mechanism by which CM-hAMSC blocked B-cell differentiation into plasma cells, we analyzed the gene expression of transcription factors responsible for the transcription of B cells, specifically B-cell CLL/lymphoma 6 (*BCL6*) and paired box 5 (*PAX-5*), and plasma cells, specifically interferon regulatory factor 4 (*IRF-4*), PR/SET domain 1 (*PRDM1*), X-box binding protein 1 (*XBP-1*), and syndecan 1 (*SDC1*/*CD138*). We observed an increase of the B-cell gene *BCL-6* in PBMC activated in the presence of CM-hAMSC, that was statistically significant at day 3 (Figure 3A), while there was no significant difference in the *PAX-5* expression (data not shown). Conversely, there was a time-dependent increased expression of plasma cells genes *IRF-4, PRDM1, XBP-1, CD138* in PBMC activated with CpG, all of which were strongly inhibited in presence of CM-AMSC (Figure 3B). Further, to support these results, we analyzed the protein expression of some transcription factors encoded by these genes (Figure 3C) by flow cytometry. At day 5, in unactivated B cells, the most highly expressed transcription factor was PAX-5. After *in vitro* CpG-stimulation, B cells increased the expression of BCL-6 and B lymphocyte induced maturation protein-1 (BLIMP-1, the transcription factor encoded by *PRDM1*), and there was a high production of IRF-4, consistent with the differentiation of B cells into ASC. When CpG stimulation was performed in presence of CM-hAMSC, we observed a slight increase of PAX-5, and no change in BCL-6. On the contrary, CM-hAMSC strongly inhibited the expression of IRF-4 and BLIMP-1, as observed at the gene level. Altogether, these data confirmed the observation that CM-hAMSC strongly blocked the terminally differentiation of B cells into plasma cells.

### Mechanism of Action of CM-hAMSC

**Figure 4:**
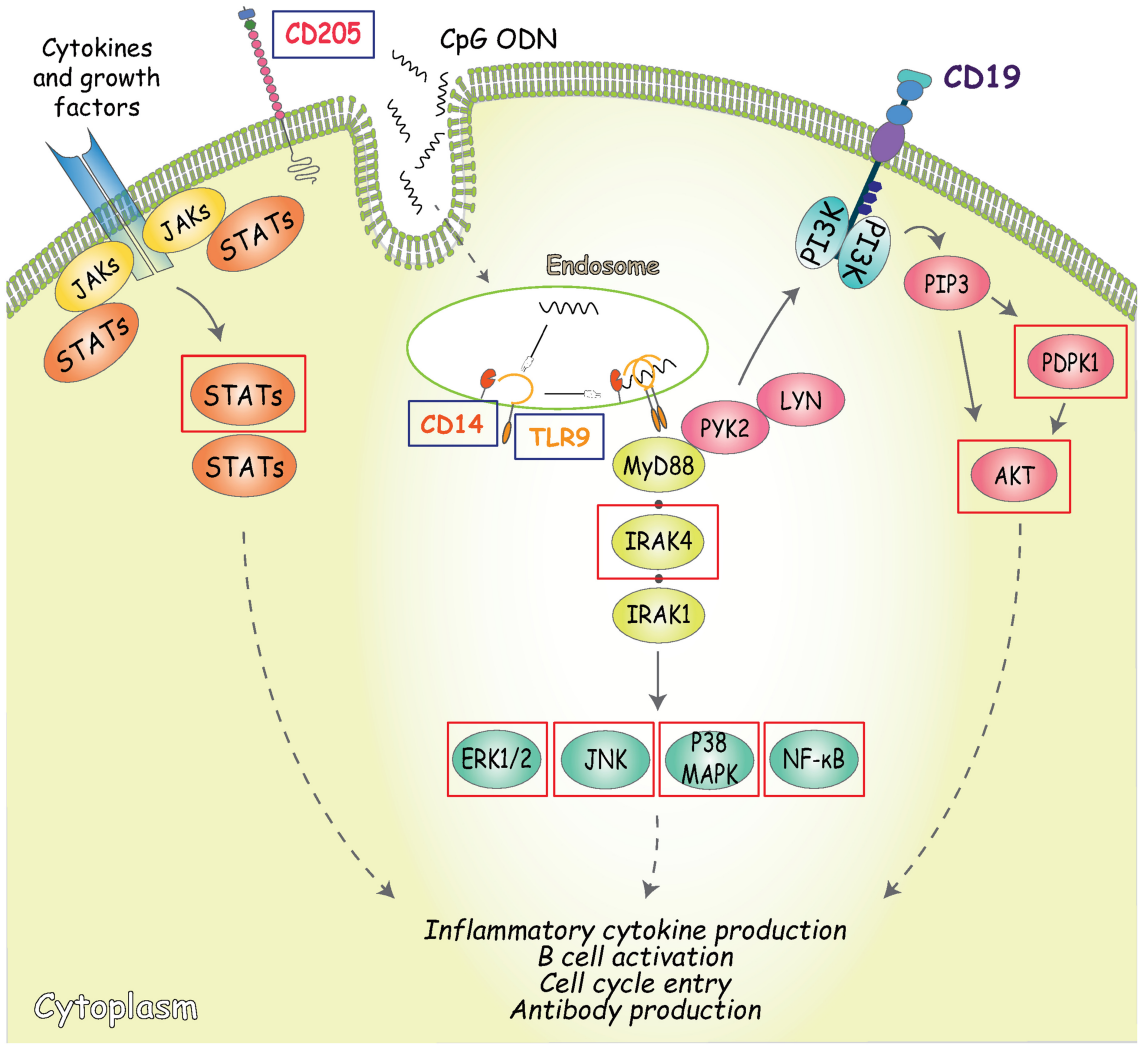
Outline of the signaling pathways activated by CpG. CD205 (DEC-205) is the main cell surface receptor for CpG and has a fundatmental role inCpG uptake and delivery to the endosomal TLR9 (53, 54). In addition to CD205, TLR9 is the primary receptor involved in CpG-signaling pathway induction, along with its co-receptor CD14 (55). TLR9 engagement in B cells is followed by the recruitment of the Myddosome complex, composed of MyD88, interleukin-1 receptor-associated kinase (IRAK)-4, and IRAK-1, where phosphorylation of IRAK1 and 4 triggers downstream signaling resulting in the activation of NF-κB signaling pathways and mitogen-activated protein kinases (MAPK) (56, 57). An alternative TLR-9-dependent pathway that also controls both early B cell activation and proliferation is represented by the phosphorylation of CD19 mediated by MyD88 (through interaction with PYK2/LYN complexes). CD19 can be regarded as one of the main regulators of PI3K activity and inducer of PDPK1 and AKT (at S473) phosphorylation (58). Arrows indicate activating phosphorylation; dashed lines indicate outcome of signaling; blue boxes indicate the CpG sensors analyzed; red boxes indicate the molecules involved in CpG signaling transduction analyzed.

**Figure 5:**
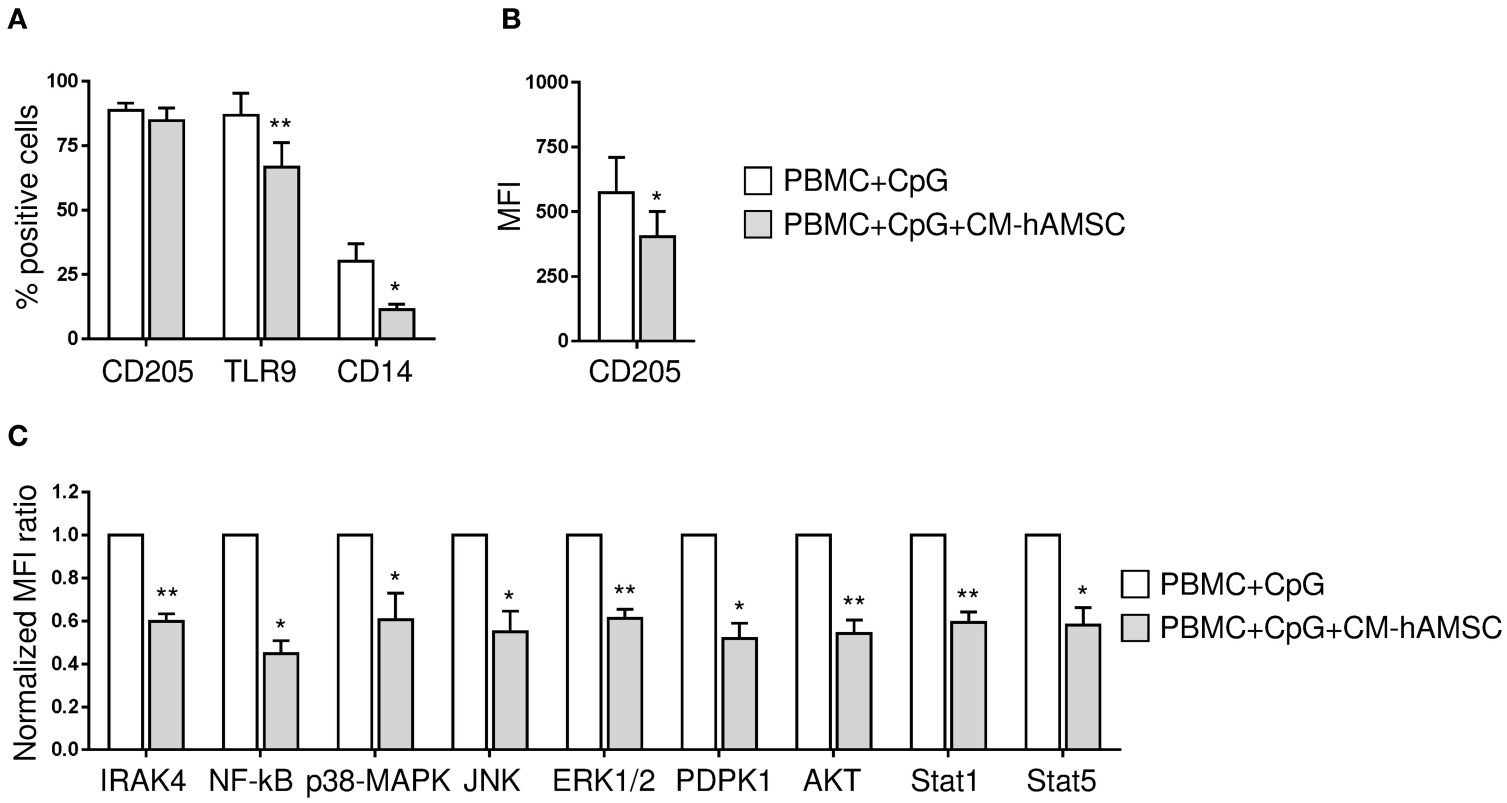
PBMC were stimulated *in vitro* with CpG in absence or presence of 100 μl CM-hAMSC. After 2 days of culture, PBMC were collected and analyzed for the expression of CpG sensors and molecules involved in CpG signaling transduction. The percentage of expression of CD205, TLR9, and CD14 on CD19^+^ cells **(A)** and the median fluorescence intensity (MFI) of CD205-positive population / MFI CD205-negative population **(B)**, was evaluated. IRAK4 and phospho NF-κB, JNK1/2, p38 MAPK, ERK1/2, AKT, PDPK1, STAT1, and STAT5 levels were measured by phosphoflow. The normalized MFI ratio is presented **(C)**, calculated as the ratio of the median of phosphoflow-positive population/median of phosphoflow negative population for each condition (PBMC+CpG and PBMC+CpG+CM-hAMSC), and then normalized against the control condition PBMC+CpG. Bars represent the mean ± SEM from at least 4 independent experiments, *p < 0.05, **p < 0.01 *vs*. PBMC+CpG.

To dissect the mechanism by which CM-hAMSC blocks B cell proliferation and differentiation, we examined the signaling pathways activated by CpG (outlined in Figure 4). Specifically, we analyzed the expression of CD205, CD14, TLR9, IRAK4, NF-κB, JNK1/2, p38 MAPK, ERK1/2, AKT, PDPK1, STAT1, and STAT5. PBMC were activated with CpG and cultured in the absence or presence of CM-hAMSC for 2 days. We then analyzed the expression of CpG sensors on CD19^+^ B cells. We found that the percentage of expression of CD205 was not affected by the presence of CM-hAMSC (Figure 5A). However, CM-hAMSC significantly decreased the cellular density expression of CD205, evaluated as median fluorescence intensity (MFI) of the receptor (CD205 MFI of PBMC+CpG = 766; CD205 MFI of PBMC+CpG+CM-hAMSC = 467, *n* = 5; *p* < 0.05; Figure 5B). Moreover, the intracellular expression of both TLR9 and CD14 was decreased in the presence of CM-hAMSC (Figure 5A). Furthermore, we found that in the presence of CM-hAMSC the expression of IRAK-4 was reduced, with a consequent reduced phosphorylation in the transactivation domain of NF-κB p65 subunit, and a reduced phosphorylation of the all three MAPK analyzed: JNK1/2, p38 MAPK, and ERK1/2 (Figure 5C). Of note, with the exception of IRAK-4, the other monoclonal antibodies used in this assay recognized the phosphorylated, therefore the activated form of the molecules. Again, we observed that CM-hAMSC reduced the expression of the phopho-PDPK1 and -AKT (Figure 5C), indicating that even this TLR-9 pathway is affected by CM-hAMSC. Finally, we found that CM-hAMSC reduced the expression of both phospho-Stat-1 and Stat-5 which play a central role in transmitting cytokine signals (Figure 5C). Altogether, these results demonstrate that CM-hAMSC is able to influence early CpG-induced B cell stimulation by reducing the expression of all the three principal CpG sensors (CD205, TLR9, and CD14), as well as the expression of IRAK-4, consequently inhibiting the entire downstream signaling pathway.

### Effect of CM-hAMSC on Cytokine Production

**Figure 6:**
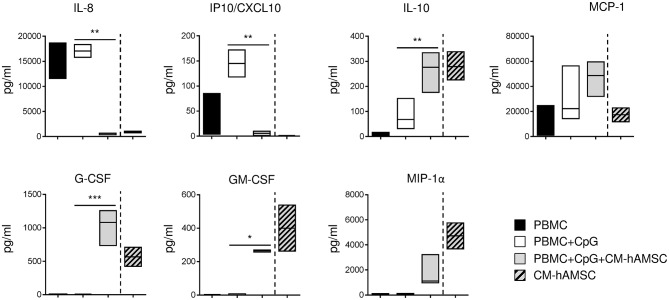
PBMC were stimulated *in vitro* with CpG and cultured in presence of 100 μl of CM-hAMSC. After 6 days of culture, the supernatant was collected and analyzed for the production of a panel of cytokines and chemokines. The amount of cytokines and chemokines present in the CM-hAMSC is represented on the right part of each plot. Floating bars (min to max) with line at median from 3 independent experiments is reported. *p < 0.05, **p < 0.01, ***p < 0.001 *vs*. PBMC+CpG.

Human B cells, in addition to their well-established role in adaptive immunity, may also regulate innate immunity through the production of cytokines and chemokines. For this reason, we investigated how CM-hAMSC could affect the production of cytokines and chemokines released in the supernatants of PBMC activated with CpG. We analyzed several cytokines and chemokines reported to be produced by PBMC activated via TLR, such as IL-8, IP10/CXCL10, MCP-1, G-CSF, GM-CSF, and MIP-1α (59), and cytokines important for plasma cell differentiation, such as IL-5, IL-2, IL-10, IL-21 (45). CpG-stimulated PBMC did not produce IL-2, IL-5, and IL-21, and we did not observed any change in presence of CM-hAMSC (data not shown). Instead, stimulated PBMC released the pro-inflammatory cytokines IL-8 and IP10/CXCL10, the production of which was completely blocked in presence of CM-hAMSC (Figure 6). Finally, CpG-stimulated PBMC also secreted MCP-1, IL-10, and low amounts of GM-CSF and MIP-1α, and the levels of these cytokines and chemokines increased in presence of CM-hAMSC. However, the variations observed could be very likely be simply ascribed to the basal concentration of the cytokines present in the CM-hAMSC (Figure 6).

### Involvement of Prostanoids in B-Cell Inhibition

**Figure 7:**
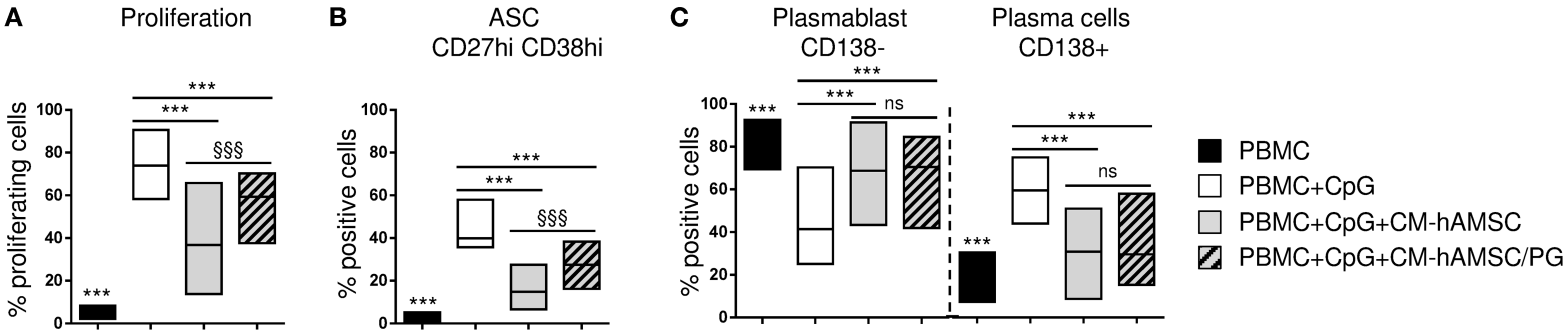
PBMC were labeled with CFSE fluorescence, stimulated *in vitro* with CpG and cultured in presence of 100 μl of CM-hAMSC or CM-hAMSC without PG (CM-hAMSC/PG). Unstimulated PBMC were used as controls. After 6 days of culture, B-cell proliferation was measured by the analysis of CFSE dilution, and calculated as percentage of CD19^+^ proliferating cells **(A)**. In addition, the percentage of antibody secreting cells (ASC, CD19^+^CD27^hi^CD38^hi^) **(B)**, and the proportion of plasmablasts (CD138^−^) and plasma cells (CD138^+^) **(C)**, were measured with flow cytometry. Floating bars (min to max) with line at median from at least 9 individual experiments is reported. ***p < 0.001 *vs*. PBMC+CpG; §§§*p* < 0.001 *vs*. PBMC+CpG+CM-hAMSC; ns, not significant.

Since we have previously shown that prostanoids are abundantly represented in CM-hAMSC and are partially responsible for the hAMSC-induced inhibition of T-cell proliferation (43), we investigated whether they could be involved in the effects observed on B cells. For this purpose, we produced CM-hAMSC in presence of indomethacin, a cyclooxygenase inhibitor, to specifically inhibit the production of prostanoids (PG), and we tested this medium (CM-hAMSC/PG) on CpG-activated PBMC. As expected, the control CM-hAMSC was able to block B-cell proliferation, instead the effect of CM-hAMSC without PG was significantly reduced (Figure 7A), suggesting a role of prostanoids in the inhibition of B-cell proliferation. We next tested the effect of CM-hAMSC/PG on B cell differentiation into ASC and plasma cells. We observed that, compared to CM-hAMSC, the block of ASC was also significantly reduced when we applied the CM-hAMSC depleted of PG (Figure 7B), further confirming the role of prostanoids affecting B cell function. In contrast, when we investigated the composition of ASC, we observed a significant (and comparable) inhibition of plasma cells with both CM-hAMSC and CM-hAMSC/PG (Figure 7C). Our results indicate that prostanoids strongly contribute to the CM-mediated modulation of CD19^+^ cell proliferation and ASC formation, but they are not responsible for the terminal inhibition of plasma cell differentiation.

## Discussion

The present study provides the first evidence that both MSC from the amniotic membrane (hAMSC) and the conditioned medium generated from their culture (CM-hAMSC), strongly suppress B-cell proliferation and differentiation, with an increase of mature B cells and a reduction of ASC formation, thus abrogating their terminal maturation into plasma cells. Indeed, we observed the strong inhibition of the expression of the proteoglycan CD138 and of *IRF-4, PRDM1*, and *XBP-1* genes, all hallmarks of terminally differentiated plasma cells. Our results point out that the mechanism by which CM-hAMSC impact B cell proliferation and differentiation is mediated by secreted factors which affect the initial B cell stimulation, consequently inhibiting the entire downstream signaling pathway. We also demonstrate that prostanoids are partially involved in these actions.

In order to study the functions of the B-cell compartment, we activated PBMC *in vitro* with the synthetic CpG oligonucleotide (ODN) 2006, an extensively characterized CpG ODN class B, among the most efficient inducers of B-cell activation, proliferation and differentiation (45, 46). We observed that not only hAMSC cultured in contact with PBMC, but also their CM were able to inhibit CpG-induced B-cell proliferation. Of note, hAMSC were not primed during CM preparation, demonstrating that suppressive factors are constitutively secreted by hAMSC without the need of activation, contrary to what described for BM-MSC which CM was not able to inhibit B cell proliferation (34). In contrast to hAMSC, some authors reported that BM-MSC and AT-MSC need to be primed with IFN-γ (32, 60) or in contact with T-cells (31, 35) to modulate B-cell proliferation. The fact that hAMSC do not require priming seems to be an intrinsic property of MSC derived from placenta, possibly linked to the role of the placenta during fetal maternal tolerance (61). B-cell differentiation has been linked to cell division (62) and thus the ability of CM-hAMSC to inhibit ASC could very likely be the consequence of the ability of CM-hAMSC to suppress B-cell proliferation. As a matter of fact, CM-hAMSC induced a dose-dependent inhibition of ASC generation that mirrored the dose dependent inhibition of CD19^+^ B cell proliferation. In addition to ASC suppression, we show the capacity of CM-hAMSC to block the terminal differentiation into CD138^+^ plasma cells. Our results are in line with those obtained by others that show a reduction of Ig production (31, 32, 34–36), and of CD138^+^ plasma cells (33, 36, 40), in presence of BM-MSC or AT-MSC. Instead, in contrast with our data, other authors have reported that BM-MSC and AT-MSC increased differentiation into plasma cells with a consequent increase of Ig production (38, 39). We also confirmed the suppression of CD138^+^ plasma cell formation by the analysis of several genes which encode transcription factors considered to be master regulators of B cell terminal differentiation. Specifically we analyzed *PRDM1*, the gene encoding for B lymphocyte induced maturation protein-1 (BLIMP-1), described to be uniquely required to drive plasmacytic differentiation and maintenance of all Ig-secreting B cells (63). Blimp-1 is necessary for the expression of J chain, X-box binding protein-1 (XBP-1), another transcriptional activator that is required for plasma cell development and function (64). In addition to *PRDM1* and *XBP-1*, we analyzed *IRF-4* that binds and activates the transcription of *prdm1*. While *blimp-1*-, *xbp-1*-, and *irf-4*-deficient mice presented severe defects in Ab production (64–66), and therefore are considered fundamental to drive plasma cell differentiation, *BCL-6* and *PAX-5* are highly expressed in mature B cells and are repressors of both *prdm1* and XBP-1 (67, 68). Our data showed that while the stimulation of PBMC with CpG induced the expression of *PRDM-1, XBP-1*, and *IRF-4*, the levels of which increased over time as previously reported (51), the presence of CM-hAMSC increased the expression of *BCL-6*, and inhibited *PRDM-1, XBP-1*, and *IRF-4*. Interestingly, XBP-1 was shown to be upregulated in CD27^hi^ cells (51), and its strong inhibition induced by CM-hAMSC was congruent with the observed suppression of CD27^hi^CD38^hi^ ASC. Moreover, the suppression of the transcription factors IRF-4 and BLIMP-1 was confirmed by flow cytometry. Thus, the interference with *PRDM-1, XBP-1*, and *IRF-4* transcription could be a way by which CM-hAMSC targets plasma cell differentiation. Analyzing the signaling pathway activated by CpG, we observed that the presence of CM-hAMSC inhibited the downstream signaling triggered by TLR9 engagement, specifically CM-hAMSC inhibited IRAK4 necessary for the activation of MAPK (JNK, p38 MAPK, ERK) and NF-κB pathways (56, 57). The inhibition of NF-κB was in line with the down-regulation of *PRDM1* observed, since NF-κB was reported to be a direct activator of *Prdm1* after TLR signals (69). In addition to the TLR9-MyD88-IRAK1/4 pathway, we analyzed the TLR9-PI3K-AKT pathway (58), and we observed a significant decreased expression of phosphorylated AKT after TLR9 stimulation in the presence of CM-hAMSC. Overall, our data suggest that factors present in the CM-hAMSC almost suppressed the entire pathway driven by CpG. Since it is well-established that CpG acts upon ligation with its specific ligand TLR9 in the endosomal compartment, we analyzed TLR9 expression. Interestingly, in presence of CM-hAMSC, the intracellular expression of TLR9 on B cells was significantly reduced. In addition, CD14 was shown to act as a coreceptor for endosomal TLR9 activation and promote the selective uptake of CpG (55). Our results show that also the expression of CD14 was reduced on B cells in CpG-activated PBMC in presence of CM-hAMSC. Finally, we observed an inhibition of the surface density expression of DEC-205/CD205, and this was of particular interest due to its key role in the uptake of class B CpG ODN, including the CpG ODN 2006 used in this study, able to shuttle CpG to endolysosomes to encounter TLR9 (53), and thus suggesting a reduction of CpG uptake. Signal transduction begins once TLR9 and its ligand are in the endolysosomal system, and although the direct relationship between the expression of TLR9 in B cells and the response mediated by CpG ODN remains elusive (70), for the first time our data shows the downregulation of both CpG-uptake sensors and TLR9 expression, and this could represent an important mechanism by which CM-hAMSC inhibit B-cell activation. Clearly, we cannot rule out that other mechanisms might be involved, such as the impairment of proteolytic cleavage of TLR9 in the endosome compartment, or the obstacle of endosomal acidification, or the modulation of adaptor molecules (e.g., TIRAP), all events of which have been described to be necessary for proper TLR9 activation, regulation, and signal transduction (46, 71–73).

Inhibiting factors from BM-MSC have been shown to be contained in secreted extracellular vescicles (EV)-enriched fractions, which were internalized by B lymphocytes and inhibit CpG-induced B cell proliferation (74, 75) and IgM, IgG, and IgA production (74). Contrarily, other authors have concluded that inhibiting factors from AT-MSC are not preferentially located in EV-enriched but rather in the EV-free (protein enriched) fractions (76). Altogether these studies corroborate our findings which highlighted a mechanism of action mediated by secreted factors, however the specific factors responsible for B cell immunomodulation are still unknown. Here, to gain an insight into the factors involved in the suppression of B-cell proliferation and differentiation induced by CM-hAMSC, we investigated the role of prostanoids, since we have previously demonstrated that they are highly found in the CM-hAMSC and are partially responsible for the hAMSC-induced inhibition of T-cell proliferation (43). While it was previously reported that MSC-derived PGE2 can promote the proliferation and differentiation of B-cells (38), our data demonstrate the involvement of prostanoids in the inhibition of B cell proliferation and ASC differentiation, in line with Su and colleagues, who described the inhibition of IgE and IgG release from activated B-cells in presence of TNF-α stimulated BM-MSC, and mediated by a cyclooxygenase 2(COX2)/PGE2 signaling pathway (77). Of note, CM-hAMSC without prostanoids was not able to block B cell proliferation and ASC formation, indicating the involvement of prostanoids in these effects. However, the block of CD138^+^ plasma cell formation was maintained. Similarly, low concentrations of CM-hAMSC were not able to inhibit either B cell proliferation or ASC formation, but were sufficient to maintain the block of plasma cell differentiation. These data revealed that other factors other than prostanoids are responsible for the block of the B cell terminal differentiation. CD138^+^ plasma-cell differentiation appears to be driven primarily by the post-activation microenvironment rather than the initial TLR-9–mediated activation event (45). Tabera and colleagues reported that they did not observe a clear maturation of B cells upon culture with CpG if plasmacytoid dendritic cells were not present in the culture (36). In line with this, when we stimulated purified B cells with CpG we observed the formation of ASC (CD27^hi^CD38^hi^), but we did not observe the complete differentiation into CD138^+^ plasma cells (data not shown). Very likely, in our culture conditions, the other immune cells present within PBMC could provide the molecules needed to generate CD138^+^ plasma cells. As a consequence, we could speculate that the factors present in CM-hAMSC responsible for the block of the B cell terminal differentiation could also act on the other immune cells involved in CD138 up-regulation. Several cytokines and growth factors have been reported to be essential to induce CD138 expression on plasma cells, including IL-2, IL-5, IL-6, IL-10, IL-21, interferon-α, hyaluronate, and human hepatocyte growth factor (45, 63). As previously reported (59), we observed that CpG-stimulated PBMC did not produce IL-2, IL-5, or IL-21, thus these cytokines very likely did not contribute to CD138^+^ plasma cell formation in these culture conditions. On the other hand, CpG-stimulated PBMC produced the pro-inflammatory chemokines IL-8 and IP10/CXCL10, the latter of which participates in plasma cell development (78), and their release was completely blocked in the presence of CM-hAMSC. Of note, IL-8 and IP10/CXCL10 inhibition is in line with the suppression of p38, JNK, ERK1/2, AKT, NF-κB, and STAT1 signaling pathway, involved in their induction (79, 80). The modulation of B cell cytokine production, in addition to the suppression of ASC, underlines the ability of CM-hAMSC to modulate diverse activities of B cells. Indeed, B cells play a well-established role in adaptive immunity by the generation of terminally differentiated ASC (20–22), and regulate innate immunity performing several immunological functions, including antigen presentation, the induction of iNKT expansion (81), and the production of multiple cytokines and chemokines (20–22).

For these reasons, B cells are a central component of many diseases, such as in autoimmune diseases including rheumatoid arthritis, systemic lupus erythematosus, or multiple sclerosis. We and others have reported the improvement of therapeutic outcome of collagen-induced arthritis (11, 12) and multiple sclerosis (11) after hAMSC infusion. Whether these improvement could be associated to a reduction of B cell proliferation and ASC formation, as observed *in vitro*, need to be investigated. Moreover, hAMSC and their CM have been successfully applied in preclinical models of lung fibrosis (3–6), where depletion of B cells represent important treatment (25, 26). In addition to the suppression of B-cell proliferation, we observed an increase of the proportion of mature naïve B cells, which is of particular interest considering the recently described role of mature naïve B cells in accelerating skin wound closure (28).

In conclusion, this study clearly demonstrates the *in vitro* activity of hAMSC and their CM on the modulation of B-cell proliferation and terminal differentiation. Our results are fundamental in order to understand the mechanism of action of amniotic cells and their CM, and even though they need to be confirmed by *in vivo* studies, they provide new insight into the possible therapeutic applications and into the mechanism of action through which they could support the restoration of tissue integrity.

## Data Availability Statement

The datasets generated for this study are available on request to the corresponding author.

## Ethics Statement

The studies involving human participants were reviewed and approved by Comitato Etico Provinciale of Brescia, Italy number NP 2243 (19/01/2016). The patients/participants provided their written informed consent to participate in this study.

## Author Contributions

MM, AM, PB, and EV carried out experiments and collected the data. MM, AM, AS, and OP designed the study, analyzed and interpreted the data, and wrote the manuscript. FS participated in drafting the manuscript. OP supervised the research and critically revised the manuscript. All authors approved the final version of the manuscript.

## Conflict of Interest

The authors declare that the research was conducted in the absence of any commercial or financial relationships that could be construed as a potential conflict of interest.

## Abbreviations

hAMSC: human amniotic mesenchymal stromal cells
CM-hAMSC: conditioned medium obtained by hAMSC culture
CM-hAMSC/PG, conditioned medium without prostanoids: obtained by hAMSC culture in presence of indomethacin.
